# Evaluation of Serum Calprotectin Levels and Their Relationship with Disease Activity in Psoriatic Arthritis and Axial Spondyloarthritis

**DOI:** 10.3390/medicina62020406

**Published:** 2026-02-20

**Authors:** Emre Ali Acar, Sadettin Uslu, Semih Gülle, Muhammet Nurullah Yiğit, Cevval Ulman, Timur Pırıldar

**Affiliations:** 1 Rheumatology Department, Tepecik Education and Research Hospital, 35530 Izmir, Turkey; dr.emrealiacar@gmail.com; 2 Division of Rheumatology, School of Medicine, Celal Bayar University, 45140 Manisa, Turkey; timur.pirildar@icloud.com; 3 Division of Rheumatology, School of Medicine, Dokuz Eylul University, 35220 Izmir, Turkey; semih.gulle@gmail.com; 4 Department of Biochemistry, Celal Bayar University School of Medicine, 45140 Manisa, Turkey; drmnyigit83@gmail.com (M.N.Y.); cevval.ulman@cbu.edu.tr (C.U.)

**Keywords:** psoriatic arthritis, axial spondyloarthritis, calprotectin, disease activity

## Abstract

*Background and Objectives*: Psoriatic arthritis (PsA) is a chronic inflammatory arthritis characterized by marked clinical heterogeneity and variable disease trajectories, underscoring the need for robust biomarkers of inflammatory burden. Serum calprotectin, a neutrophil- and monocyte-derived protein, has been proposed as a surrogate marker of active inflammation in inflammatory arthritis due to its close association with innate immune activation. In this study, we compare serum calprotectin levels among patients with PsA, axial spondyloarthritis (AxSpA), and healthy controls and evaluate their association with disease activity. *Materials and Methods*: This single-center, cross-sectional study included 123 patients with PsA, 119 patients with AxSpA, and 77 healthy controls. Serum calprotectin levels were measured by enzyme-linked immunosorbent assay, and their associations with disease activity were evaluated using correlation, multivariable regression, and receiver operating characteristic analyses. *Results*: Serum calprotectin levels were significantly higher in PsA and AxSpA patients compared with healthy controls (*p* < 0.001 for both) and were higher in PsA than in AxSpA (*p* = 0.022). In PsA, serum calprotectin levels showed significant correlations with ASDAS-CRP, DAS28-CRP, and DLQI, but not with CRP or ESR. In contrast, in AxSpA, calprotectin showed only a weak association with CRP and was not related to disease activity indices. In multivariable analysis, serum calprotectin was independently associated with ASDAS-CRP in PsA (B = 0.704, *p* = 0.003), but not in AxSpA. Receiver operating characteristic analysis demonstrated that serum calprotectin discriminated high disease activity in PsA with an area under the curve of 0.669 (95% CI: 0.563–0.775; *p* = 0.003). *Conclusions*: Serum calprotectin levels are elevated in patients with PsA and are associated with disease activity, supporting its potential role as a biomarker in this condition. In contrast, serum calprotectin does not appear to reflect disease activity in AxSpA, suggesting disease-specific differences in its clinical utility.

## 1. Introduction

Psoriatic arthritis (PsA) is a chronic immune-mediated inflammatory arthritis associated with psoriasis, encompassing a broad clinical spectrum that includes peripheral arthritis, axial involvement, enthesitis, dactylitis, and extra-articular manifestations [1]. This phenotypic heterogeneity translates into substantial inter-individual variability in disease course, structural damage, and treatment response, thereby complicating disease monitoring and the implementation of individualized therapeutic strategies. Axial spondyloarthritis (AxSpA), another prototypic inflammatory rheumatic disease predominantly affecting the axial skeleton, similarly relies on composite clinical indices and acute-phase reactants for disease activity assessment, which may not fully capture the inflammatory burden across all patients [2].

Although imaging modalities and validated activity indices have improved disease assessment, conventional systemic inflammatory markers—erythrocyte sedimentation rate (ESR) and C-reactive protein (CRP)—show limited sensitivity and specificity for active inflammation in a meaningful subset of patients. Notably, normal CRP values may be observed despite clinically active disease, highlighting the need for biomarkers that better reflect ongoing innate immune activation and tissue-level inflammation [3,4,5].

Calprotectin (S100A8/S100A9; also known as MRP8/14) is a myeloid-derived heterodimer released predominantly by activated neutrophils and monocytes/macrophages and is considered a surrogate of leukocyte activation and migration [6]. Owing to its relative stability in serum and feasibility of quantification by enzyme-linked immunosorbent assay (ELISA), serum calprotectin has emerged as a potential biomarker in inflammatory arthritis, with reported associations with synovial inflammation and clinical disease activity in several disease settings [7]. Importantly, in conditions characterized by prominent synovial and entheseal inflammation, serum calprotectin may provide incremental information beyond conventional acute-phase reactants for disease monitoring and, potentially, treatment-response assessment. However, evidence regarding the clinical utility of serum calprotectin in PsA remains limited, and head-to-head comparisons across PsA, AxSpA, and healthy populations are scarce. Moreover, the extent to which serum calprotectin aligns with standardized disease activity measures within these patient groups has not been fully clarified.

Therefore, this study aimed (i) to evaluate serum calprotectin levels in patients with PsA compared with patients with AxSpA and healthy controls, and (ii) to investigate the association between serum calprotectin concentrations and disease activity measures. By delineating the potential role of calprotectin as a biomarker in these conditions, we sought to inform more accurate disease monitoring and support individualized management strategies in PsA.

## 2. Materials and Methods

This study was designed as a single-center, cross-sectional observational study comparing patients with PsA, AxSpA, and healthy controls. A total of 123 consecutive PsA patients fulfilling the Classification Criteria for Psoriatic Arthritis (CASPAR) [8] and 119 AxSpA patients meeting the Assessment of Spondyloarthritis International Society (ASAS) classification criteria [9] were recruited from the rheumatology clinic of a university hospital between March 2024 and March 2025. The control group consisted of 77 healthy adult volunteers without a history of inflammatory or autoimmune disease.

Patients with concomitant autoimmune or inflammatory rheumatic diseases, inflammatory bowel disease, a history of malignancy or serious infection, severe renal and/or hepatic insufficiency, or conditions limiting the ability to complete self-reported questionnaires were excluded. Pregnant individuals and participants younger than 18 years of age were also excluded from the study.

Participants completed validated self-reported outcome measures, including the Bath Ankylosing Spondylitis Disease Activity Index (BASDAI), the Bath Ankylosing Spondylitis Functional Index (BASFI), the Ankylosing Spondylitis Disease Activity Score (ASDAS), and the Health Assessment Questionnaire (HAQ). Patients in the PsA group additionally completed the Psoriatic Arthritis Quality of Life (PsAQoL) questionnaire and the Dermatology Life Quality Index (DLQI), while patients in the AxSpA group completed the Ankylosing Spondylitis Quality of Life (ASQoL) questionnaire. Disease activity was categorized according to ASDAS-CRP cut-off values as inactive disease (<1.3), low disease activity (1.3–<2.1), high disease activity (2.1–<3.5), and very high disease activity (≥3.5).

Venous blood samples were obtained from patients into clot-activator tubes without anticoagulant. The samples were centrifuged at 3000 rpm for 15 min to separate the serum, and the serum aliquots were stored at −80 °C until analysis. Calprotectin concentrations in serum samples were analyzed using the ELISA method. ELISA washing procedures were performed using an automated washer (BioTek ELx50, BioTek Instruments Inc., Highland Park, Winooski, VT, USA), and absorbance readings were obtained using an ELISA reader (BioTek Epoch, BioTek Instruments Inc., Highland Park, Winooski, VT, USA). Serum calprotectin levels were measured using a Calprotectin ELISA Kit (Elabscience, Houston, TX, USA) according to the procedures specified in the manufacturer’s protocol, with a 1:50 dilution. The initial results were multiplied by 50 to obtain final values. Ng/mL values were divided by 1000 to convert them to µg/mL. Intra-assay coefficient of variation (%CV) values were reported as 5.91% at a concentration of 4.91 ng/mL, and inter-assay %CV values were reported as 5.34% at 5.24 ng/mL. The kit sensitivity was 10.94 ng/mL. The measurement range of the kit was 1.56–100 ng/mL. The Control 1 value range was 21.08–34.95 ng/mL, and the Control 2 value range was <1.47 ng/mL. In all four ELISA runs, both Control 1 and Control 2 were detected within the expected value ranges.

### 2.1. Ethical Considerations

The study was approved by the Manisa Celal Bayar University School of Medicine Local Ethics Committee (Date: 12 July 2023; Decision No: 20.478.486/1921) and was conducted in accordance with the principles of the Declaration of Helsinki and its subsequent amendments. Written informed consent was obtained from all participants prior to inclusion in the study.

### 2.2. Statistical Analysis

All statistical analyses were carried out using IBM SPSS Statistics for Windows, version 25.0 (IBM Corp., Armonk, NY, USA). Continuous data were summarized as mean ± standard deviation, depending on data distribution, whereas categorical variables were reported as counts and percentages. Data distribution was evaluated using both graphical methods (histograms and Q–Q plots) and appropriate normality assessments. Comparisons of continuous variables among groups were performed using the Kruskal–Wallis test, followed by the Mann–Whitney U test for pairwise comparisons when applicable. Categorical variables were analyzed using the chi-square test. Associations between variables were examined using Spearman’s rank correlation analysis. Multivariable linear regression models were constructed to determine independent predictors of serum calprotectin levels. The discriminatory performance of serum calprotectin for identifying high disease activity was evaluated using receiver operating characteristic (ROC) curve analysis. All analyses were two-sided, and statistical significance was defined as a *p*-value < 0.05.

## 3. Results

A total of 123 patients with PsA, 119 patients with AxSpA, and 77 healthy controls were included. In the PsA group, 77 patients (62.6%) were female and 46 (37.3%) were male, whereas the AxSpA group showed a marked male predominance (91 males [76.4%] and 28 females [23.5%]). The healthy control group comprised 40 females (51.9%) and 37 males (48.1%). Sex distribution differed significantly among groups (*p* < 0.001), driven primarily by the male predominance in the AxSpA group. Mean age was 47.66 ± 11.42 years in PsA, 43.93 ± 10.57 years in AxSpA, and 44.40 ± 5.74 years in controls, with PsA patients being significantly older (*p* = 0.014).

Mean serum calprotectin levels were 3.85 ± 2.24 µg/mL in PsA, 3.25 ± 1.99 µg/mL in AxSpA, and 1.51 ± 0.58 µg/mL in healthy controls. Serum calprotectin concentrations were significantly higher in both PsA and AxSpA compared with controls (*p* < 0.001 for both), and they were also higher in PsA than in AxSpA (*p* = 0.022). In contrast, mean ESR and CRP levels did not differ significantly between PsA and AxSpA (ESR: 26.81 ± 19.49 vs. 28.95 ± 19.99 mm/h, *p* = 0.395; CRP: 0.69 ± 0.93 vs. 1.37 ± 2.99 mg/dL, *p* = 0.052). Table 1 summarizes the baseline characteristics of the study population.

In PsA, serum calprotectin showed moderate positive correlations with ASDAS-CRP, DAS28-CRP, and DLQI, while no significant correlations were observed with CRP or ESR (Table 2). In multivariable linear regression analysis adjusted for age, sex, CRP, and ESR, serum calprotectin remained independently associated with ASDAS-CRP (B = 0.704, *p* = 0.003), whereas CRP and ESR were not independently associated. The overall model was statistically significant (R^2^ = 0.092, *p* = 0.044), indicating that ASDAS-CRP was the primary determinant of serum calprotectin levels in PsA.

In AxSpA, serum calprotectin exhibited a weak but significant correlation with CRP, but showed no significant associations with ASDAS-CRP, BASDAI, BASFI, or functional and quality-of-life measures (Table 3). Multivariable regression analysis in AxSpA did not identify any independent predictors of serum calprotectin levels; the overall model was not significant (R^2^ = 0.034, *p* = 0.563) (Table 4).

**Figure 1:**
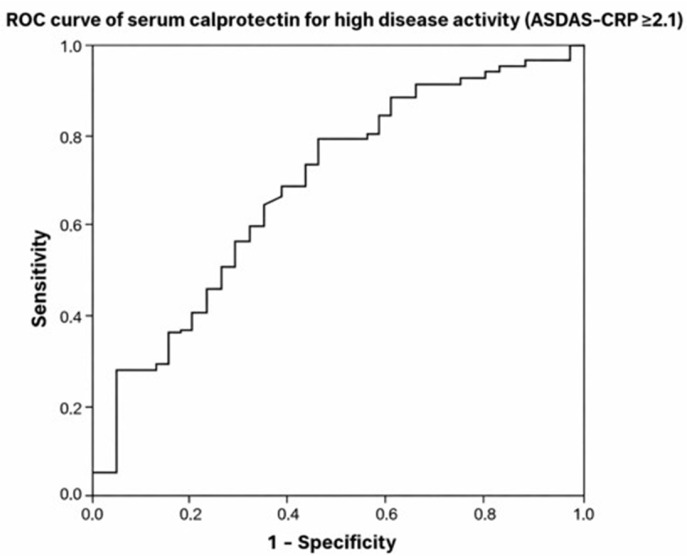
Receiver operating characteristic (ROC) curve of serum calprotectin for discriminating high disease activity in patients with PsA. High disease activity was defined as ASDAS-CRP ≥ 2.1. The area under the curve (AUC) was 0.669 (95% CI: 0.563–0.775; *p* = 0.003).

**Figure 2:**
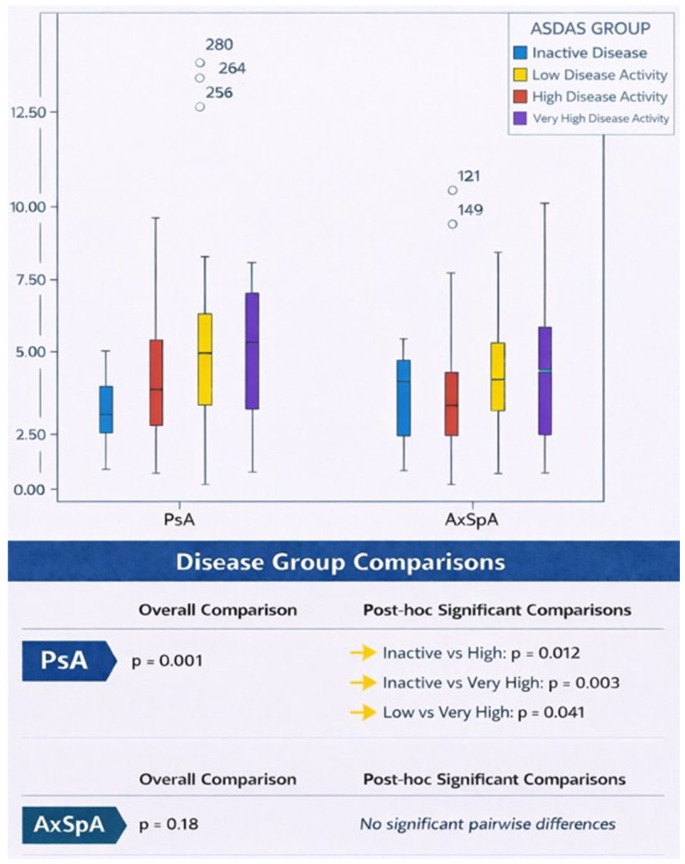
Serum calprotectin levels across ASDAS disease activity categories in patients with PsA and AxSpA. Box plots show median and interquartile range; whiskers indicate minimum and maximum values, and circles denote outliers. Overall group differences were assessed using the Kruskal–Wallis test, followed by Bonferroni-adjusted Mann–Whitney U post hoc comparisons where applicable.

ROC analysis demonstrated that serum calprotectin discriminated high disease activity (ASDAS-CRP ≥ 2.1) in PsA with an area under the curve of 0.669 (95% CI: 0.563–0.775; *p* = 0.003). A calprotectin cut-off of approximately 2.3 µg/mL yielded a sensitivity of 80.5% and a specificity of 38.9% for identifying high disease activity (Figure 1). Serum calprotectin levels differed significantly across ASDAS disease activity categories in PsA, whereas no significant differences were observed in AxSpA (Figure 2).

## 4. Discussion

In this study, serum calprotectin levels were significantly higher in patients with PsA and AxSpA compared with healthy controls, supporting its role as a marker of systemic inflammation in inflammatory rheumatic diseases. Calprotectin levels were also significantly higher in PsA than in AxSpA. Importantly, calprotectin levels in healthy controls and patient groups were comparable to those reported in previous studies, supporting the external validity of our findings [10].

The distinct correlation patterns observed between serum calprotectin and disease activity measures in PsA and AxSpA highlight the phenotype-dependent nature of this biomarker. In PsA, serum calprotectin showed significant associations with composite disease activity indices and with dermatology-related quality of life as assessed by DLQI, indicating that it may reflect aspects of systemic inflammatory burden in this disease. In contrast, in AxSpA, calprotectin demonstrated only a weak association with CRP and was not significantly related to ASDAS-CRP or patient-reported disease activity measures. The independent association between serum calprotectin and ASDAS-CRP in PsA, but not with conventional acute-phase reactants such as CRP or ESR, further supports the notion that calprotectin captures dimensions of inflammatory burden beyond systemic acute-phase responses and may better reflect composite disease activity. Notably, this independent relationship was not observed in AxSpA, reinforcing the concept that the clinical relevance of calprotectin varies according to disease phenotype. Although the discriminative performance of calprotectin for identifying high disease activity in PsA was moderate, the statistically significant AUC suggests that serum calprotectin may serve as a valuable adjunctive biomarker, particularly in patients with active disease despite normal conventional inflammatory markers.

In the literature, calprotectin is described as an alarmin belonging to the damage-associated molecular pattern family, released predominantly from activated neutrophils and monocytes, and is considered to reflect both synovial and systemic inflammation. In a large cohort study by Jarlborg et al. [10], serum calprotectin levels were shown to correlate significantly with disease activity indices, including DAS28 and ASDAS, in patients with rheumatoid arthritis and AxSpA; however, this association was weaker in PsA. This observation has been attributed to the heterogeneity of inflammatory burden and the multisystemic nature of PsA. In our study, serum calprotectin levels likewise demonstrated a positive correlation with ASDAS. This finding suggests that calprotectin may capture inflammatory activity even in the absence of elevated classical acute-phase reactants such as CRP and ESR. Indeed, elevated CRP is not observed in approximately half of patients with both AxSpA and PsA, further supporting the potential value of calprotectin as an alternative biomarker.

Previous research has demonstrated positive correlations between serum calprotectin levels and conventional inflammatory markers, as well as disease activity indices, including CRP, ESR, BASDAI, and ASDAS, particularly in AxSpA [10,11,12,13,14]. In line with these observations, our study identified a significant association between serum calprotectin levels and ASDAS-CRP, most prominently in PsA patients. This finding is clinically relevant, as calprotectin may reflect inflammatory activity even in patients with normal acute-phase reactants, a well-recognized limitation of CRP and ESR in both PsA and AxSpA.

Beyond reflecting disease activity, calprotectin has also been proposed as a biomarker for predicting treatment response and structural progression. Several studies have reported that serum calprotectin levels decrease following effective anti-inflammatory interventions, including tumor necrosis factor inhibitors and structured exercise programs, suggesting its potential value as an early indicator of therapeutic response [11,14,15,16]. Moreover, elevated baseline calprotectin levels have been independently associated with radiographic progression and new syndesmophyte formation in axial disease, highlighting a possible role in predicting long-term structural outcomes [14,17].

Despite these promising findings, the diagnostic and prognostic utility of serum calprotectin remains a subject of debate. In particular, serum calprotectin appears to be less sensitive than fecal calprotectin for detecting intestinal inflammation. While fecal calprotectin more accurately reflects subclinical gut inflammation and the risk of inflammatory bowel disease in spondyloarthritis, serum calprotectin primarily represents systemic inflammatory burden [18]. Accordingly, combined assessment of serum and fecal calprotectin may offer complementary information in selected clinical scenarios.

In PsA specifically, multiple studies have demonstrated that serum calprotectin levels are significantly higher in patients with arthritis compared with both healthy controls and individuals with psoriasis without joint involvement, suggesting a closer association with musculoskeletal inflammation than with cutaneous disease activity [19,20]. Calprotectin levels have been shown to correlate positively with disease activity measures such as Disease Activity Index for Psoriatic Arthritis (DAPSA), swollen joint count, pain scores, and imaging-based indicators of synovitis, including ultrasonography [21,22,23]. Furthermore, higher calprotectin concentrations have been reported in patients with polyarticular disease compared with those with mono- or oligoarticular involvement, indicating that systemic inflammatory burden may vary according to disease pattern [19,24].

Several studies have also suggested that lower baseline calprotectin levels are associated with a higher likelihood of achieving minimal disease activity or remission, whereas elevated baseline levels may predict relapse under biologic therapy and outperform traditional markers such as CRP or ESR in this regard [20,23,25]. Additionally, correlations between serum calprotectin levels and ultrasonographic synovitis support its potential role in detecting subclinical inflammation, particularly in early PsA [21,24,25].

Nevertheless, not all studies have confirmed the superiority of calprotectin over conventional inflammatory markers. Some large-scale analyses have reported weak or inconsistent correlations between calprotectin and disease activity, particularly in patients with low disease activity states [19,21]. The heterogeneity of reported findings may be attributable to differences in study design, patient selection, disease phenotype distribution, sample size, comorbid inflammatory conditions, and assay methodologies.

The ROC analysis demonstrated only a moderate discriminative performance of serum calprotectin for identifying high disease activity in PsA. This finding indicates that calprotectin should not be considered a stand-alone diagnostic tool. However, in complex, multisystem diseases such as PsA, biomarkers reflecting a single inflammatory pathway rarely achieve high discriminatory accuracy. The consistent associations observed across multiple clinical domains suggest that calprotectin may provide complementary information regarding systemic inflammatory burden when interpreted alongside clinical assessment.

Calprotectin levels in PsA have been reported to be higher, particularly in patients with polyarticular involvement, suggesting that the magnitude of systemic inflammatory burden may vary according to the pattern of musculoskeletal involvement. Furthermore, the literature indicates that calprotectin levels correlate more strongly with articular disease activity than with the extent of cutaneous involvement, supporting its role as a more specific marker of joint inflammation. Consistent with these observations, studies by Farouk et al. [26] and Li et al. [20] demonstrated that elevated serum calprotectin levels in patients with PsA were significantly associated with DAPSA scores, swollen joint counts, and ultrasonographic evidence of synovitis.

Several limitations should be considered when interpreting the findings of this study. The cross-sectional design precludes causal inference and does not permit assessment of temporal changes in serum calprotectin in relation to disease activity fluctuations or structural progression. Composite indices such as ASDAS-CRP include patient-reported pain components that may be influenced by non-inflammatory factors, including mechanical spinal pathology, degenerative changes, or central sensitization, potentially leading to overestimation of inflammatory burden in some individuals.

Furthermore, detailed phenotypic and treatment-related characterization of the study cohorts was not systematically available. Information on PsA clinical phenotype (peripheral, axial, or mixed), extent of spinal involvement, disease duration, body mass index, presence of enthesitis or dactylitis, and stratification by ongoing therapies (including NSAID and biologic use) could not be uniformly incorporated into multivariable models. These variables may influence systemic inflammatory biomarkers, and their absence limits adjustment for potential confounding.

Although ASDAS-CRP was employed to allow standardized assessment across PsA and AxSpA groups, it is not a PsA-specific composite index and may not fully capture the heterogeneity of peripheral musculoskeletal involvement typical of PsA. The lack of DAPSA scoring represents an additional limitation. Moreover, NSAID therapy, which is common in spondyloarthritis, and subclinical intestinal inflammation—frequently reported in these conditions may also contribute to circulating calprotectin levels. Fecal calprotectin was not assessed, and although patients with known inflammatory bowel disease were excluded, the potential contribution of subclinical intestinal inflammation to circulating calprotectin levels cannot be entirely ruled out. Systematic gastrointestinal evaluation (endoscopy or fecal calprotectin assessment) was not performed, and therefore intestinal contributions to systemic calprotectin levels cannot be entirely excluded.

Finally, differences in mean calprotectin levels between groups may partly reflect unmeasured clinical or treatment-related factors rather than purely inflammatory disease burden. Nevertheless, serum calprotectin reflects innate immune activation rather than pain perception alone, and its consistent association with multiple disease activity domains in PsA supports the biological plausibility of a true inflammatory signal. The findings should therefore be interpreted as demonstrating an association rather than a causal relationship.

Despite these limitations, the relatively large sample size and inclusion of both PsA and AxSpA cohorts strengthen the internal consistency of the findings. Future longitudinal studies incorporating detailed phenotypic profiling, treatment stratification, imaging data, gastrointestinal assessment, and PsA-specific composite indices are required to further define the biomarker role of calprotectin.

## 5. Conclusions

This cross-sectional study demonstrates that serum calprotectin levels are significantly elevated in patients with PsA and are associated with disease activity as assessed by ASDAS-CRP, supporting its role as a marker of systemic inflammatory burden in PsA. In contrast, no significant association was observed between calprotectin levels and disease activity in AxSpA, suggesting phenotype-dependent utility. Owing to its feasibility and close link to innate immune activation, serum calprotectin may represent a valuable adjunctive tool for disease monitoring in PsA; however, prospective longitudinal studies are required to define its predictive value and to establish its role in routine clinical practice.

## Figures and Tables

**Table 1:** Demographic, clinical, and laboratory characteristics of the study groups.

Variable	PsA (*n* = 123)	AxSpA (*n* = 119)	Controls (*n* = 77)	*p* Value
Sex, ***n*** (%)				<0.001
Female	77 (62.6)	28 (23.5)	40 (51.9)	
Male	46 (37.3)	91 (76.4)	37 (48.1)	
Age, years	47.66 ± 11.42	43.93 ± 10.57	44.40 ± 5.74	0.014
ESR, mm/h	26.81 ± 19.49	28.95 ± 19.99	9.39 ± 1.75	<0.001
Calprotectin, µg/mL	3.85 ± 2.24	3.25 ± 1.99	1.51 ± 0.58	<0.001
CRP, mg/dL	0.69 ± 0.93	1.37 ± 2.99	0.15 ± 0.02	<0.001
ASDAS-CRP	2.67 ± 1.03	2.83 ± 1.04	–	–
DAS28-CRP	2.71 ± 0.98	–	–	–
HAQ	0.67 ± 0.28	0.51 ± 0.56	–	–
PsAQoL	8.36 ± 6.44	–	–	–
DLQI	5.77 ± 7.11	–	–	–
ASQoL	–	7.36 ± 5.70	–	–
BASDAI	-	4.39 ± 2.19	–	–
BASFI	-	3.02 ± 2.49	–	–

Continuous variables are presented as mean ± standard deviation. Group comparisons for continuous variables were performed using the Kruskal–Wallis test, followed by the Mann–Whitney U test for pairwise comparisons when appropriate. Categorical variables were compared using the chi-square test. Bonferroni correction was applied for multiple comparisons, with statistical significance set at *p* < 0.0167. Abbreviations: ESR: Erythrocyte sedimentation rate; CRP: C-reactive protein; ASDAS-CRP: Ankylosing Spondylitis Disease Activity Score using C-reactive protein; DAS28-CRP: Disease Activity Score in 28 joints using C-reactive protein; HAQ: Health Assessment Questionnaire; PsAQoL: Psoriatic Arthritis Quality of Life; DLQI: Dermatology Life Quality Index; ASQoL: Ankylosing Spondylitis Quality of Life; BASDAI: Bath Ankylosing Spondylitis Disease Activity Index; BASFI: Bath Ankylosing Spondylitis Functional Index.

**Table 2:** Spearman correlation analysis between serum calprotectin and clinical, laboratory, and disease activity parameters in patients with PsA.

Variable	Spearman’s ρ	*p* Value
ASDAS-CRP	0.285	0.001
DAS28-CRP	0.341	<0.001
DLQI	0.288	0.001
HAQ	0.128	0.159
PsAQoL	0.164	0.069
CRP	0.074	0.413
ESR	0.010	0.917

Spearman’s rank correlation coefficients (ρ) were calculated for patients with PsA (*n* = 123).

**Table 3:** Spearman correlation analysis between serum calprotectin and clinical, laboratory, and disease activity parameters in patients with AxSpA.

Variable	Spearman’s ρ	*p* Value
CRP	0.248	0.006
ASDAS-CRP	0.162	0.079
BASFI	0.018	0.843
BASDAI	−0.001	0.987
ASQoL	0.026	0.782
HAQ	0.003	0.976
ESR	0.113	0.220

Spearman’s rank correlation coefficients (ρ) were calculated for patients with AxSpA (*n* = 119).

**Table 4:** Multivariable linear regression analysis of factors associated with serum calprotectin levels in patients with psoriatic arthritis.

Variable	PsA (*n* = 123) B (SE)	*p* Value	AxSpA (*n* = 119) B (SE)	*p* Value
Age (years)	−0.003 (0.019)	0.878	0.016 (0.018)	0.383
Sex (male/female)	0.572 (0.436)	0.192	0.244 (0.448)	0.587
ASDAS-CRP	0.704 (0.229)	0.003	0.284 (0.216)	0.191
CRP (mg/dL)	−0.260 (0.272)	0.341	−0.011 (0.087)	0.900
ESR (mm/h)	−0.015 (0.013)	0.251	0.002 (0.011)	0.877

Model summary: PsA: R^2^ = 0.092; Adjusted R^2^ = 0.053; F = 2.36; *p* = 0.044, AxSpA: R^2^ = 0.034; Adjusted R^2^ = −0.009; F = 0.78; *p* = 0.563. Values are unstandardized regression coefficients (B) with standard errors (SE). Multivariable linear regression models were adjusted for age, sex, ASDAS-CRP, CRP, and ESR.

## Data Availability

The data presented in this study are available upon request from the corresponding author.

## Abbreviations

The following abbreviations are used in this manuscript:

ASDAS-CRP	Ankylosing Spondylitis Disease Activity Score using C-reactive protein
ASQoL	Ankylosing Spondylitis Quality of Life questionnaire
AxSpA	Axial spondyloarthritis
BASDAI	Bath Ankylosing Spondylitis Disease Activity Index
BASFI	Bath Ankylosing Spondylitis Functional Index
CRP	C-reactive protein
DAPSA	Disease Activity Index for Psoriatic Arthritis
DAS28-CRP	Disease Activity Score in 28 joints using C-reactive protein
DLQI	Dermatology Life Quality Index
ESR	Erythrocyte sedimentation rate
HAQ	Health Assessment Questionnaire
PsA	Psoriatic arthritis
PsAQoL	Psoriatic Arthritis Quality of Life questionnaire
